# A phylogenomically informed five-order system for the closest relatives of land plants

**DOI:** 10.1016/j.cub.2022.08.022

**Published:** 2022-10-24

**Authors:** Sebastian Hess, Shelby K. Williams, Anna Busch, Iker Irisarri, Charles F. Delwiche, Sophie de Vries, Tatyana Darienko, Andrew J. Roger, John M. Archibald, Henrik Buschmann, Klaus von Schwartzenberg, Jan de Vries

**Affiliations:** 1 Institute for Zoology, University of Cologne, Zülpicher Str. 47b, 50674 Cologne, Germany; 2 Department of Biochemistry and Molecular Biology, Dalhousie University, 5850 College St., Halifax NS B3H 4R2, Canada; 3 University of Goettingen, Institute for Microbiology and Genetics, Department of Applied Bioinformatics, Goldschmidtstr. 1, 37077 Goettingen, Germany; 4 University of Goettingen, Campus Institute Data Science (CIDAS), Goldschmidstr. 1, 37077 Goettingen, Germany; 5 Cell Biology and Molecular Genetics, University of Maryland-College Park, College Park, MD, USA; 6 University of Applied Sciences Mittweida, Faculty of Applied Computer Sciences and Biosciences, Section Biotechnology and Chemistry, Molecular Biotechnology, Technikumplatz 17, 09648 Mittweida, Germany; 7 Universität Hamburg, Institute of Plant Science and Microbiology, Microalgae and Zygnematophyceae Collection Hamburg (MZCH) and Aquatic Ecophysiology and Phycology, Ohnhorststr. 18, 22609 Hamburg, Germany; 8 University of Goettingen, Goettingen Center for Molecular Biosciences (GZMB), Department of Applied Bioinformatics, Goldschmidtstr. 1, 37077 Goettingen, Germany

**Keywords:** conjugating green algae, conjugatophyceae, Charophyta, streptophyte algae, multicellularity, phylogenomics, plant terrestrialization, plant evolution

## Abstract

The evolution of streptophytes had a profound impact on life on Earth. They brought forth those photosynthetic eukaryotes that today dominate the macroscopic flora: the land plants (Embryophyta).^1^ There is convincing evidence that the unicellular/filamentous Zygnematophyceae—and not the morphologically more elaborate Coleochaetophyceae or Charophyceae—are the closest algal relatives of land plants.^2^, ^3^, ^4^, ^5^, ^6^ Despite the species richness (>4,000), wide distribution, and key evolutionary position of the zygnematophytes, their internal phylogeny remains largely unresolved.^7^^,^^8^ There are also putative zygnematophytes with interesting body plan modifications (e.g., filamentous growth) whose phylogenetic affiliations remain unknown. Here, we studied a filamentous green alga (strain MZCH580) from an Austrian peat bog with central or parietal chloroplasts that lack discernible pyrenoids. It represents *Mougeotiopsis calospora* PALLA, an enigmatic alga that was described more than 120 years ago^9^ but never subjected to molecular analyses. We generated transcriptomic data of *M. calospora* strain MZCH580 and conducted comprehensive phylogenomic analyses (326 nuclear loci) for 46 taxonomically diverse zygnematophytes. Strain MZCH580 falls in a deep-branching zygnematophycean clade together with some unicellular species and thus represents a formerly unknown zygnematophycean lineage with filamentous growth. Our well-supported phylogenomic tree lets us propose a new five-order system for the Zygnematophyceae and provides evidence for at least five independent origins of true filamentous growth in the closest algal relatives of land plants. This phylogeny provides a robust and comprehensive framework for performing comparative analyses and inferring the evolution of cellular traits and body plans in the closest relatives of land plants.

## Highlights

• Comprehensive phylogenomic analyses for 46 taxonomically diverse Zygnematophyceae
• Five-order system for the Zygnematophyceae, the closest relatives of land plants
• Filamentous and pyrenoid-lacking *Mougeotiopsis* sits in a deep clade of unicells
• Evidence for at least five independent origins of true filamentous growth

## Graphical abstract

**Figure:**
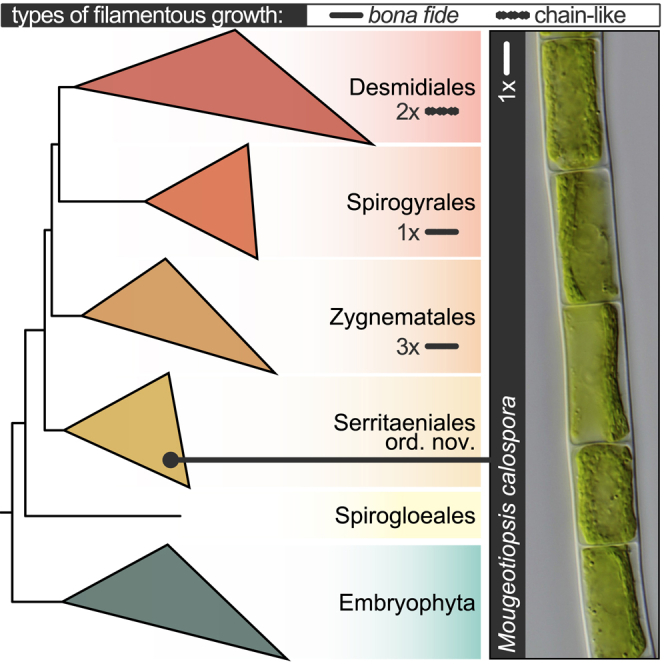

## Teaser

Hess et al. use comprehensive phylogenomic analyses and present a five-order system for the Zygnematophyceae. They place the filamentous and pyrenoid-lacking *Mougeotiopsis* among unicellular zygnematophytes. Based on this framework, they propose at least five independent origins of true filamentous growth for the closest relatives of land plants.

## Results and discussion

### Morphology and phylogenetic position of a filamentous zygnematophyte without pyrenoids

**Figure 1:**
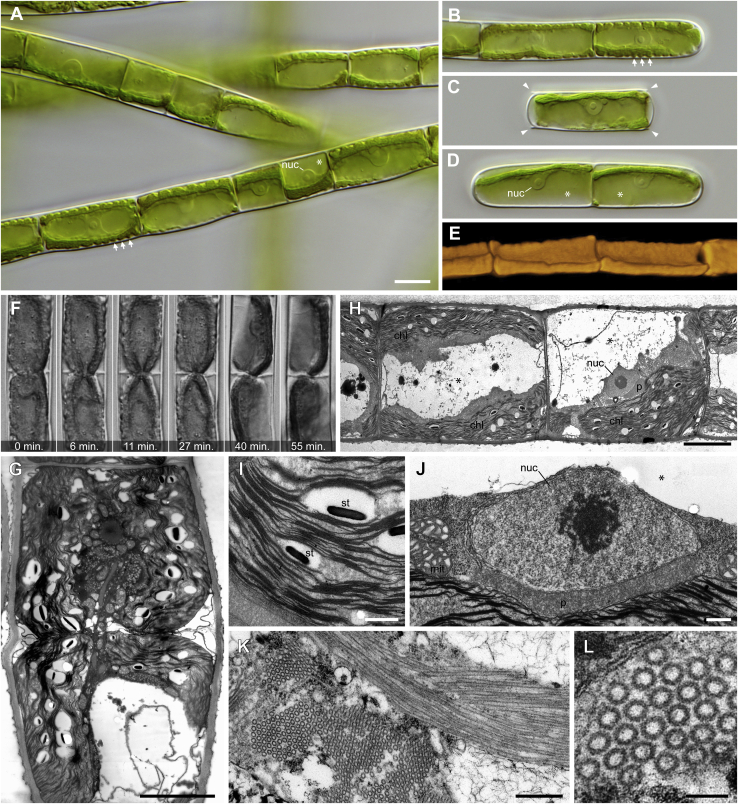
Morphology, cell division, and ultrastructure of *Mougeotiopsis calospora* strain MZCH580 (A) Filaments with cells of varying length; differential interference contrast (DIC). Note the indented chloroplast margins (arrows), the prominent nuclei (nuc) and the large vacuoles (asterisk). (B) Filament with rounded tip; DIC. (C) Single cell after fragmentation with cell wall remnants (arrowheads); DIC. (D) Two-celled filament with smooth tips; DIC. Note the prominent nuclei (nuc) and the large vacuoles (asterisks). (E) Three-dimensional reconstruction of the chloroplasts based on their autofluorescence; confocal microscopy. (F) Time series of a dividing cell shows ingrowing cross wall; DIC. (G) Ultrathin section through a dividing cell reveals the ingrowing cell wall (see plasma membrane) and the chloroplast in division. (H) Ultrathin section through vegetative filament showing the position of the nucleus (nuc), peroxisome (p), chloroplasts (chl) and vacuoles (asterisks). (I) Ultrathin section of starch grains (st) between the thylakoids of the chloroplast. (J) Ultrathin section of the nucleus (nuc) with nucleolus, the large, elongate peroxisome (p), and mitochondria (mit). The vacuolar space is marked by the asterisk. (K) Ultrathin section of bundled macrotubules in cross section (left) and longitudinal section (right). (L) Detail of macrotubules in cross section. Scale bars 10 μm in (A) (applies also for B–D); 5 μm in (G) and (H); 500 nm in (I)–(K); 100 nm in (L). See also Figures S1, S2, and S4.

Strain MZCH580 forms unbranched filaments with smooth cell walls and rounded tips (Figures 1A and 1B). Infolded cross walls (“replicate walls”) or rhizoids known from some filamentous zygnematophytes^10^ were not observed in our cultures. The filaments of strain MZCH580 tend to fragment as the cultures age, but cells divide and grow back into new filaments when fresh medium is added (Figures 1C and 1D). Interphase cells are 10–15 μm wide (mean = 12 μm, n = 40) and 12–55 μm long (mean = 22 μm, n = 80), and usually contain a single chloroplast. The chloroplast lacks visible pyrenoids and has a variable shape ranging from an off-center straight plate (Figure 1D) to a more parietal morphology, like a channel or half-pipe (Figures 1A and 1B). The 3D reconstruction of confocal fluorescence data reveals a common intermediate morphology (Figure 1E). The lateral sides of half-pipe-shaped chloroplasts display clear indentations, which are rare in filamentous green algae with chloroplasts of similar morphology (Figures 1A and 1B, arrows)—*Entransia fimbriata* (Klebsormidiophyceae), for example, has fimbriate or lobed chloroplasts, but of much more irregular morphology.^11^ The nucleus is spherical (4–6 μm in diameter, n = 40) with a prominent central nucleolus (1–3 μm in diameter, n = 40), and always closely associated with the chloroplast (Figures 1A and 1D; nuc). Both chloroplast and nucleus are surrounded by a thin sheath of cytoplasm and opposed to or surrounded by a large vacuole (Figure 1D; asterisks).

Cell division is intercalary and involves the centripetal formation of a cross wall (Figure 1F; Videos S1 and S2). We did not observe any phragmoplast-like structure as known from many streptophyte algae.^12^, ^13^, ^14^ Instead, ingrowing cell wall material seemed to pinch off the chloroplast (Figure 1F and Videos S1 and S2), which is corroborated on the ultrastructural level (Figure 1G). It appears that the chloroplast does not divide before the inset of cytokinesis, and that the cell division in strain MZCH580 largely depends on furrowing (cleavage, thus centripetal cell wall ingrowth). However, we cannot exclude the existence of a phragmoplast and our ultrastructural data of late stages of cytokinesis seem compatible with phragmoplast-like structures as known from many streptophyte algae, including other zygnematophytes (e.g., *Spirogyra* and *Mougeotia*^12^^,^^13^).

Video S1. Cytokinesis in *Mougeotiopsis calospora*: Centripetal cell division, related to Figure 1 The movie shows that cytokinesis is dominated by centripetal cleavage. Note the rotating chloroplasts and the migrating nuclei. Imaging was performed at six frames per minute, shown as 10 FPS

Video S2. Cytokinesis in *Mougeotiopsis calospora*, related to Figure 1 A second movie to illustrate the different phenotypes of cell division; see description for Video S1.

Our ultrastructural data confirm that the chloroplasts of strain MZCH580 lack pyrenoids but contain numerous lentiform starch grains (up to ∼1 μm) interspersed between the thylakoids (Figures 1H and 1I). This is a very unusual chloroplast configuration. Pyrenoids are found in all other known zygnematophytes (and most green algae) and are considered important compartments for carbon concentration. That said, hornworts have frequently gained and lost pyrenoids—a phenomenon that does not correlate with atmospheric CO_2_ concentration or lifestyle changes.^15^ *Mougeotiopsis* appears to compensate for the lack of pyrenoid-based carbon concentration by an extremely high expression of homologs of *ribulose-1,5-bisphosphate carboxylase/oxygenase small subunit 2* (*rbcS2*) and *rubisco activase* (*rca*); in fact, with transcripts per million (TPM) values of 44002 and 15238, they were, respectively, the highest and fourth highest expressed transcript in the whole transcriptome. In contrast, in the transcriptomes of the pyrenoid-bearing alga *Mougeotia* sp. MZCH240, *rbcs* and *rca* homologs never ranged among the top 100 most abundant transcripts (see de Vries et al.^16^ and Fürst-Jansen et al.^17^). The ecophysiological consequences of the absence of pyrenoids in *Mougeotiopsis* are currently obscure.

Other noteworthy ultrastructural characteristics of strain MZCH580 are a giant peroxisome situated between the nucleus and the chloroplast (Figure 1J), and the occurrence of macrotubules (∼44 nm in diameter; 44.02 nm ± 2.4 nm, n = 446) in cells with incomplete cytokinesis likely promoted by environmental factors (Figures 1K, 1L, and S1); the occurrence of macrotubules has been described in land plant tissues—for example, in cells of root tips but with a distinct mean diameter^18^ (35 nm). A single peroxisome of similar localization was also reported for Klebsormidiophyceae such as *Klebsormidium*, *Hormidiella*, and *Streptosarcina*,^19^, ^20^, ^21^, ^22^ and the Zygnematophyceae *Zygogonium*,^23^ suggesting that this is a rather widespread character in streptophyte algae. However, the filamentous zygnematophytes *Mougeotia*, *Spirogyra*, and *Zygnema* contain numerous, much smaller peroxisomes, which do not exceed 1 μm in our TEM sections (Figure S2).

Based on taxonomic comparisons (see Table 1 and STAR Methods for details), we apply the name *Mougeotiopsis calospora* to strain MZCH580. However, as we did not observe any sexual processes (conjugation, flagellated gametes), zoospores, or aplanospores in our cultivated material, the suspected affinity to the zygnematophytes remained uncertain. While analysis of the *rbcL* gene (coding for the large chain of ribulose-1,5-bisphosphate carboxylase/oxygenase) placed strain MZCH580 within the streptophytes, a robust phylogenetic placement was not possible. To scrutinize the phylogenetic position of strain MZCH580, we generated RNA-seq data by Illumina sequencing and performed a *de novo* transcriptome assembly. The resulting transcriptome has a completeness of 96.3% (benchmarked universal 272 single-copy orthologs) and contains 52,188 predicted open reading frames (ORFs). We built a comprehensive multigene dataset of 326 conserved proteins (see STAR Methods) from streptophyte algae, land plants, and select chlorophyte algae as outgroup, with 84 taxa in total (see species and deposited data in STAR Methods). Our phylogenomic inferences with a sophisticated site-heterogeneous model of protein sequence evolution (LG+PMSF(C60)+F+Γ) resulted in a well-supported phylogeny, whose overall topology is in line with current knowledge about streptophyte evolution (cf. Figure S3 and One Thousand Plant Transcriptomes Initiative^6^). To scrutinize this, we performed an approximately unbiased (AU) test under the best-fit model LG+C60+F+Γ with 10,000 multiscale bootstrap replicates. Our dataset rejected the topology of the One Thousand Plant Transcriptomes Initiative^6^ (AU test p = 0.000). This, however, only concerned some relationships within Desmidiales, and neither their monophyletic arrangement nor any other aspect of the gross topology, thus also having no effect on any trait inferences below. Strain MZCH580 groups within the Zygnematophyceae with full nonparametric bootstrap support and forms a deep-branching lineage with the unicellular *Serritaenia* sp. (strain CCAC 0155) and “*Mesotaenium endlicherianum*” (strain SAG 12.97). Hence, strain MZCH580, referred to as *Mougeotiopsis calospora* hereafter, is clearly distinct from other filamentous genera (*Mougeotia*, *Spirogyra*, *Zygnema*, and *Zygnemopsis*), and represents a new lineage of zygnematophytes with filamentous growth.

**Table 1:** Five-order taxonomy of the Zygnematophyceae

**Order Serritaeniales**

S.Hess & J.de Vries *ord. nov.* Diagnosis: comprises unicells and filaments with smooth sidewalls, cells with axial or parietal chloroplasts, and simple cell walls (no pores and ornamentations), phylogenetically closely related to the type species (*Serritaenia testaceovaginata*; *rbc*L MW159377). Type:*Serritaeniaceae* S.Hess & J.de Vries *fam. nov.* Family Serritaeniaceae S.Hess & J.de Vries *fam. nov.* Diagnosis: with characteristics of order Serritaeniales; unicells and filaments with smooth sidewalls, cells with axial or parietal chloroplasts, and simple cell walls (no pores and ornamentations), embedded or not in the mucilage. Type:*Serritaenia* A.Busch & S.Hess 2021. Comment: Currently the Serritaeniales includes a single family, the Serritaeniaceae with the genera *Serritaenia* and *Mougeotiopsis*.

**Order Zygnematales**

Bessey *emend*. S.Hess & J.de Vries Emended description: Comprises unicells and unbranched and uniseriate filaments with smooth side walls, cells with stellate, plate- or ribbon-like chloroplasts and simple cell walls (no pores and ornamentations), phylogenetically closely related to strains SAG 698-1a (Genbank transcriptome shotgun assembly GFYA00000000). Type:*Zygnema* C.Agardh, 1817, *nom. et typ. cons.* Comment: Currently the Zygnematales includes a single family Zygnemataceae with the genera *Cylindrocystis, Mesotaenium* (current assumption, pending discovery of type species)*, Mougeotia, Zygnema,* and *Zygnemopsis*. No culture is available from the type species of *Zygnema*.

**Order Desmidiales**

Bessey *emend*. S.Hess & J.de Vries Emended description: Comprises unicells and chain-like filaments. Cell walls and morphologies of diverse complexity, including the "placoderm desmids" with cell wall pores, ornamentations and clear isthmus, and species with smooth cell walls and without isthmus. Phylogenetically closely related to strain *Desmidium aptogonum* (RNA-seq ERX2100155). Type:*Desmidium* C.Agardh ex Ralfs, 1848. Comment: Currently the Desmidiales includes a single family Desmidiaceae with the genera *Bambusina, Closterium, Cosmarium, Cosmocladium, Desmidium, Euastrum, Micrasterias, Netrium, Nucleotaenium, Onychonema, Penium, Phymatodocis, Planotaenium, Pleurotaenium, Staurastrum, Staurodesmus, Xanthidium*, and more. No culture is available from the type species of *Desmidium*.

**Order Spirogyrales**

Clements *emend*. S.Hess & J.de Vries Emended description: Comprises filaments with smooth side walls, cells with one or more helical chloroplast and smooth cell walls without pores or ornamentation. Phylogenetically closely related to strain *Spirogyra pratensis* strain MZCH10213 (RNA-seq data: NCBI BioProject PRJNA543475, TSA GICF00000000). Type:*Spirogyra* Link, 1820, *nom. cons.* Comment: Currently the Spirogyrales includes only the genus *Spirogyra*. The closely related genus *Sirogonium* Kützing may also belong to this order, but this needs to be confirmed by phylogenomic studies. No culture is available from the type species of *Spirogyra*. The order Spirogyrales was originally validated by Clements (1909: 12); his description specified “Typically one-celled or filamentous algae, without zoospores; sexual reproduction by the conjugation of similar gametes; two fungous families.” No fungi are currently included in this order.

### Phylogenomics support a five-order taxonomy of the Zygnematophyceae

**Figure 2:**
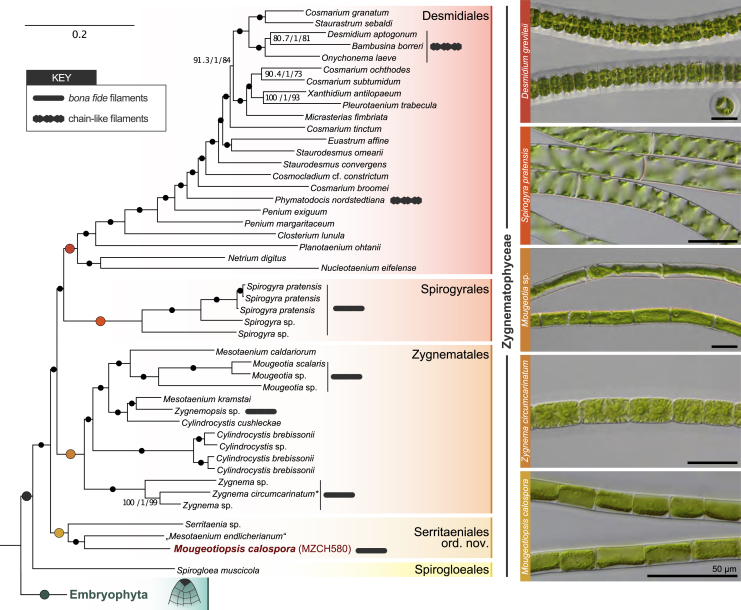
Position of strain MZCH580 in a well-resolved zygnematophycean phylogeny based on 326 genes Section of the phylogenomic tree limited to zygnematophytes and embryophytes. Support values from three analyses (SH-aLRT/aBayes/nonparametric bootstrapping) are shown at the corresponding branches, except for branches with maximum support (marked by dots); large colored dots correspond to the (full) support recovered for the higher-order clades labeled on the right. The Zygnematophyceae comprise five deep-branching clades, which are here defined as orders. Gray symbols highlight zygnematophytes that form chain-like filaments (see micrograph of *Desmidium*) and *bona fide* filaments (see micrographs of *Spirogyra*, *Mougeotia*, *Zygnema*, and *Mougeotiopsis*); scale bars in all micrographs are 50 μm. Scale bar for phylogeny is 0.2 expected substitutions per site. The entire phylogenomic tree with all streptophyte taxa is shown in Figure S3. Asterisk: a recent study by Feng et al.^24^ found that SAG698-1a might be *Z. cylindricum* instead of *Z. circumcarinatum*.

Previous phylogenies based on single (or few) marker genes have suggested that the traditional taxonomic separation into the two orders Desmidiales and Zygnematales does not reflect the evolutionary relationships of the Zygnematophyceae.^7^^,^^8^ Yet, the taxonomy of this important algal class remains unresolved, in part due to the lack of robust phylogenetic data. Our multigene phylogeny clearly demonstrates that the Zygnematales as previously defined (all filamentous members plus unicells that are not placoderm desmids) are paraphyletic. Instead, the Zygnematophyceae comprise at least five deep-branching clades that we feel can be treated at the level of orders (Figure 2).

We introduce a new, phylogenomically informed five-order taxonomy of the Zygnematophyceae, by reinterpreting existing ordinal names and introducing a new order for *Mougeotiopsis* and its unicellular relatives (see Table 1). The Serritaeniales *ord. nov.* currently comprises the name-giving genus *Serritaenia* (unicells with a plate-like chloroplast and a mostly aerophytic life style^25^), the genome-sequenced strain SAG 12.97 (often referred to as “*Mesotaenium endlicherianum*”^26^; unicells with half-pipe-like chloroplasts and an aquatic lifestyle) and *Mougeotiopsis calospora*, strain MZCH580. Although these species differ markedly in growth form (unicells versus filaments), their chloroplasts are all characterized by indented or undulated margins^25^^,^^26^ that are otherwise rare in zygnematophytes. Yet, *Mougeotiopsis calospora* is the only known zygnematophyte that lacks pyrenoids.

Our data corroborate the position of the Spirogloeales, consisting of the unicellular *Spirogloea muscicola* (formerly *Spirotaenia muscicola*), as sister lineage to all other Zygnematophyceae.^26^ For the remaining part of the phylogenomic tree, we redefine three traditional orders. The Zygnematales are now limited to a morphologically diverse clade comprising unicellular zygnematophytes currently assigned to *Cylindrocystis* and *Mesotaenium*, plus three distinct branches of filamentous members (*Mougeotia*, *Zygnema*, and *Zygnemopsis*); the recovered topology demonstrates the polyphyly of the unicellular genera belonging to that order (*Cylindrocystis* and *Mesotaenium*), which require a taxonomic revision in the future. Chloroplasts of the Zygnematales are either stellate (*Cylindrocystis*, *Zygnema*, and *Zygnemopsis*) or ribbon/plate-like with smooth margins (*Mesotaenium* and *Mougeotia*).

The *Spirogyra* species with their characteristic helical chloroplasts form another, deep-branching clade, which is here defined as Spirogyrales Clements 1909 (Figure 2 and Table 1). This order was initially introduced to include algae of yellow-green appearance (including *Spirogyra*) and some fungal families.^27^ We limit the concept of the Spirogyrales to those zygnematophycean algae that form the sister clade of the Desmidiales in our phylogeny. The latter order mainly comprises symmetric unicells with a pronounced central constriction (isthmus) and ornamented cell walls. However, at the base of the clade containing these typical placoderm desmids are three genera (*Netrium*, *Nucleotaenium*, and *Planotaenium*), which display a much simpler morphology (no cell wall ornamentations and no isthmus) and were formerly classified with the Zygnematales (in the family Mesotaeniaceae).^28^ Interestingly, the same arrangement was previously recovered by combined analyses of three genes (nuclear SSU rRNA, *rbcL*, and chloroplast LSU rRNA),^29^ and is here confirmed by phylogenomics. It appears that the desmids with elaborate cell shapes and complex cell walls (e.g., *Cosmarium*, *Penium*, *Micrasterias*, and *Xanthidium*) descended from unicellular ancestors with a simpler structure. Hence, the genera *Netrium*, *Nucleotaenium*, and *Planotaenium* are here formally included in the order Desmidiales. The internal phylogeny and taxonomy of the Desmidiales, however, needs to be resolved by extended taxon sampling in the future, as many classically recognized desmid genera (e.g., *Cosmarium*, *Penium*, and *Staurodesmus*) are not monophyletic.

### On the unicellularity of the ancestral zygnematophyte

Our robust phylogenetic framework of the zygnematophytes now enables comparisons of species in an evolutionary context; thus assessment of evolutionary scenarios with great confidence are feasible. It is remarkable that the majority of zygnematophycean species are unicellular,^30^ as most of their streptophyte relatives (Embryophyta, Coleochaetophyceae, Charophyceae, Klebsormidiophyceae, and Chlorokybophyceae) display some kind of multicellularity, from sarcinoids to three-dimensional tissues.^31^ However, some zygnematophycean lineages exhibit more developmental complexity such as the formation of filaments, sometimes even with rhizoids or branched cells.^31^^,^^32^ Traditionally these filamentous members have been bundled in the family Zygnemataceae,^28^ but a close relationship of them was not recovered in previous phylogenies.^7^^,^^8^

**Figure 3:**
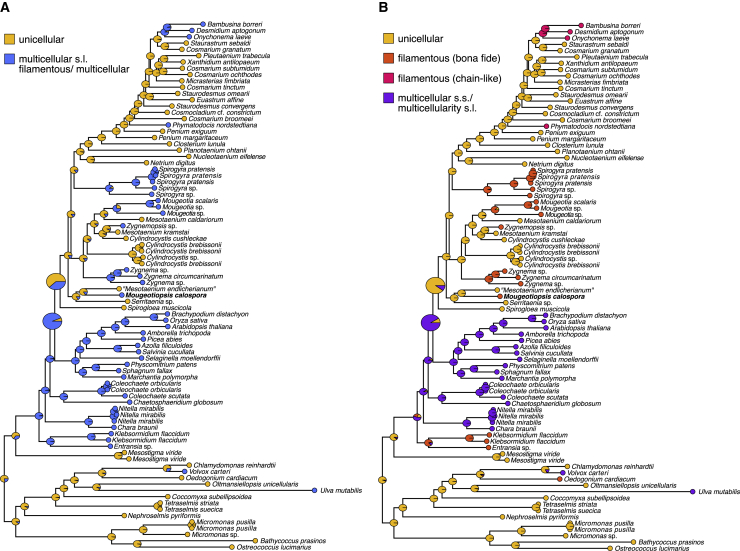
Ancestral character state reconstruction for unicellular or multicellular (including filamentous) growth characters (A and B) Growth types were coded as either a simplistic (A) two- and more nuanced (B) four- character state distributions to reflect different levels of complexity regarding the possibilities/hypotheses for the homology of growth types: yellow, unicellular; blue, multicellular *sensu lato* (including filamentous growth); orange, *bona fide* filamentous growth; pink, chain-like filaments (desmids); and purple, multicellular growth *sensu stricto*.

Our fully supported phylogenomic tree reveals at least five separate lineages that contain true filaments, found in three orders (Figure 2): *Spirogyra* (Spirogyrales), *Mougeotia, Zygnema, Zygnemopsis* (all Zygnematales), and *Mougeotiopsis* (Serritaeniales). Other filamentous taxa (e.g., *Temnogametum iztacalense* and *Zygogonium ericetorum*) await genomic/transcriptomic sequencing and phylogenomic placement^33^^,^^34^ The cells of all these filamentous species have straight and relatively simple cell walls, no central constrictions, and display an intimate cell-cell contact (i.e., typical cross walls)—yet without plasmodesmata.^35^ At the same time, there are also filamentous desmids (e.g. *Desmidium*, *Bambusina*, *Onychonema,* and *Phymatodocis*^36^), which differ markedly from the aforementioned lineages in their cellular details and filament morphology (see also Hall et al.^37^). The cells of *Desmidium*, *Bambusina*, *Onychonema*, and *Phymatodocis* display the typical characters of desmid cells (e.g., central constriction and cell wall ornamentation) and rather appear as cell chains. Together with the fact that the filamentous desmids are nested within the unicellular desmids, it is conceivable that there are distinct types of filamentous growth in the Zygnematophyceae, which evolved independently; we account for this possibility in our analyses (see Figure 3 and below). The Zygnematophyceae as a whole are nested within a clade of mostly multicellular streptophytes, the Phragmoplastophyta, with the most morphologically elaborate (the Embryophyta) as sister clade. Previous studies have therefore noted that the streamlined body plans of extant zygnematophytes—down to unicellularity—might have arisen by reductive evolution from a morphologically more complex ancestor.^5^^,^^38^, ^39^, ^40^, ^41^ Based on our current phylogeny, it seems most parsimonious that the last common ancestor of the zygnematophytes was unicellular—thus having already experienced a reduction in its body plan. This scenario goes along with five independent origins of *bona fide* filaments; the alternative would require at least seven losses of multicellularity.

In an attempt to infer the body plan of the common ancestor of zygnematophytes, we performed ancestral character state reconstructions (ACSR) with various data coding strategies concerning the types of multicellularity (Figure 3). Irrespective of how the growth types were coded, a unicellular zygnematophyte ancestor was consistently inferred by our analyses, albeit with varying support (posterior probability [PP] = 0.58–0.93). Hence, we infer up to five tentative independent origins of true filamentous growth, and two additional independent origins of chain-like filaments (in the Desmidiales) (unicellular ancestors have PP = 0.80–1.00); under this scenario, the last common ancestor of the Zygnematophyceae and land plants was likely filamentous or multicellular (PP = 0.91–0.93), whereas the last common ancestor of Zygnematophyceae was likely unicellular (PP = 0.58–0.89). Given the effect of character coding in these analyses, we conclude that expanding our knowledge about the homology of the various types of multicellular and filamentous body plans in the green algae is essential.

Filamentous growth as observed in the Zygnematophyceae can be considered the least elaborate type of multicellularity.^42^ Yet, the cellular and molecular traits underpinning this growth type remain obscure. The multiple growth type transitions in the zygnematophytes are consistent with parallel evolution from a common molecular machinery, but the relative simplicity of filamentous growth renders convergent evolution equally plausible. The hypothetical unicellular lynchpin at the base of the Zygnematophyceae is an attractive hypothesis: it could explain why zygnematophytes lack plasmodesmata (e.g., Brunkard and Zambryski^35^), why the cross walls often look distinct from other streptophytes, and perhaps even why the group as such returned to a cleavage-like cell division mechanism (see Buschmann and Zachgo^14^). Future research on the different filamentous lineages will need to establish a deeper understanding of the molecular machinery underpinning their common morphology.

In addition, recent culture-based efforts to explore terrestrial zygnematophytes indicated a high diversity of unicellular lineages,^43^ which are not yet covered by genomic/transcriptomic sequencing and might change the evolutionary picture. Biased taxon sampling is indeed a serious problem for ACSR,^44^^,^^45^ and thus genomic sequencing of further zygnematophytes is an important task for the future. The fossil record for Zygnematophyceae is sparse. Several of the ordinal lineages of Zygnematophyceae are potentially several hundreds of millions of years old (estimations based on molecular clock results presented in Morris et al.^46^). Hence, important information might be obscured by extinction events and new discoveries of living or fossil taxa could easily lead to new interpretations. For now, our phylogenomic data demonstrate that the zygnematophytes comprise multiple transitions of their body plan, and also enable the selection of relevant species for comparative cell biological research.

### Conclusion

The identification of the Zygnematophyceae as the sister lineage to land plants was surprising, in part because of their relatively simple body plans. The study of zygnematophycean trait evolution is a challenge because of their species richness, diverse morphologies, and unresolved phylogeny. We have provided a phylogenomic backbone and a congruent classification system for the closest algal relatives of land plants. Looking at algal growth types through the lens of phylogenomics reveals dynamic emergence and formation of filamentous and unicellular growth among the Zygnematophyceae—traits whose evolutionary history might also feature reductive evolution from a more complex ancestor of Zygnematophyceae and land plants.

## STAR★Methods

### Key resources table

**Table:** 

REAGENT or RESOURCE	SOURCE	IDENTIFIER
**Critical commercial assay**s		

DNAse I	Thermo Fisher, Waltham, MA, USA	N/A
NEB mRNA stranded Library preparation kit	New England Biolabs, Beverly, MA, USA	N/A
Trizol	Thermo Fisher, Waltham, MA, USA	N/A

**Deposited data**		

Alignment	This study	https://doi.org/10.5281/zenodo.6805950
*Amborella trichopoda* genome	Amborella genome project^47^	https://phytozome.jgi.doe.gov/pz/portal.html#!info?alias=Org_Atrichopoda
*Arabidopsis thaliana* genome TAIR V10	TAIR^48^	http://www.arabidopsis.org
*Azolla filiculoides* genome	Li et al.^49^	https://www.fernbase.org
*Bambusina borreri* CCAC 0045 transcriptome, 1KP Code QWFV	Carpenter et al.^50^	http://www.onekp.com/public_data.html
*Bathycoccus prasinos* genome	Moreau et al.^51^	https://phycocosm.jgi.doe.gov/Batpra1/Batpra1.info.html
*Brachypodium distachyon*	The International Brachypodium Initiative^52^	https://phytozome-next.jgi.doe.gov/info/Bdistachyon_v3_1
*Chaetophaeridium globosum* SAG26.98 transcriptome	Cooper and Delwiche^53^	https://figshare.com/articles/dataset/Green_algal_transcriptomes_for_phylogenetics_and_comparative_genomics/1604778
*Chara braunii* S276 genome	Nishiyama et al.^54^	https://bioinformatics.psb.ugent.be/orcae/overview/Chbra
*Chlamydomonas reinhardtii* genome v5.5	Merchant et al.^55^; Blaby et al.^56^	https://phytozome.jgi.doe.gov/pz/portal.html#!info?alias=Org_Creinhardtii
*Closterium lunula* M2156 transcriptome, 1KP Code DRFX	Carpenter et al.^50^	http://www.onekp.com/public_data.html
*Coccomyxa subellipsoidea* genome v2.0	Blanc et al.^57^	https://phytozome.jgi.doe.gov/pz/portal.html#!info?alias=Org_CsubellipsoideaC_169
*Coleochaete scutata* SAG50.90 transcriptome	de Vries et al.^58^	https://www.ncbi.nlm.nih.gov/Traces/wgs/wgsviewer.cgi?val=GFXZ00000000
*Coleochaete orbicularis* transcriptome	Ju et al.^59^	https://www.ncbi.nlm.nih.gov/Traces/wgs/wgsviewer.cgi?val=GBSL01&search=GBSL01000000&display=scaffolds
*Coleochaete orbicularis* transcriptome	Cooper and Delwiche^53^	https://figshare.com/articles/dataset/Green_algal_transcriptomes_for_phylogenetics_and_comparative_genomics/1604778
*Cosmarium broomei* CCAC 0143 transcriptome, 1KP Code HIDG	Carpenter et al.^50^	http://www.onekp.com/public_data.html
*Cosmarium granatum* CCAC 0137 transcriptome, 1KP Code MNNM	Carpenter et al.^50^	http://www.onekp.com/public_data.html
*Cosmarium ochthodes* M1384 transcriptome, 1KP Code HJVM	Carpenter et al.^50^	http://www.onekp.com/public_data.html
*Cosmarium subtumidum* M3067 transcriptome, 1KP Code WDGV	Carpenter et al.^50^	http://www.onekp.com/public_data.html
*Cosmarium tinctum* M2301 transcriptome, 1KP Code BHBK	Carpenter et al.^50^	http://www.onekp.com/public_data.html
*Cosmocladium* cf. *constrictum* ASW 07118 transcriptome, 1KP Code RQFE	Carpenter et al.^50^	http://www.onekp.com/public_data.html
*Cylindrocystis brebissonii* M2213 transcriptome, 1KP Code YOXI	Carpenter et al.^50^	http://www.onekp.com/public_data.html
*Cylindrocystis brebissonii* M2853/M2213 transcriptome, 1KP Code YLBK	Carpenter et al.^50^	http://www.onekp.com/public_data.html
*Cylindrocystis brebissonii* M2853 transcriptome, 1KP Code RPGL	Carpenter et al.^50^	http://www.onekp.com/public_data.html
*Cylindrocystis cushleckae* M2158 transcriptome, 1KP Code JOJQ	Carpenter et al.^50^	http://www.onekp.com/public_data.html
*Cylindrocystis* sp. M3015 transcriptome, 1KP Code VAZE	Carpenter et al.^50^	http://www.onekp.com/public_data.html
*Desmidium aptogonum* ASW 07112 transcriptome, 1KP Code DFDS	Carpenter et al.^50^	http://www.onekp.com/public_data.html
*Entransia* sp. transcriptome	Cooper and Delwiche^53^	https://figshare.com/articles/dataset/Green_algal_transcriptomes_for_phylogenetics_and_comparative_genomics/1604778
*Euastrum affine* ASW 07012 transcriptome, 1KP Code GYRP	Carpenter et al.^50^	http://www.onekp.com/public_data.html
*Klebsormidium flaccidum* UTEX 321 transcriptome	Cooper and Delwiche^53^	https://figshare.com/articles/dataset/Green_algal_transcriptomes_for_phylogenetics_and_comparative_genomics/1604778
*Klebsormidium flaccidum* SAG2307 transcriptome	de Vries et al.^58^	https://www.ncbi.nlm.nih.gov/Traces/wgs/wgsviewer.cgi?val=GFXY00000000
*Marchantia polymorpha* genome v3.1	Bowman et al.^60^	https://phytozome.jgi.doe.gov/pz/portal.html#!info?alias=Org_Mpolymorpha
*Mesotaenium braunii* (*Serritaenia* sp.) M2214 transcriptome, 1KP Code WSJO	Carpenter et al.^50^	http://www.onekp.com/public_data.html
*Mesotaenium caldariorum* SAG 648-1 transcriptome, 1KP Code HKZW	Carpenter et al.^50^	http://www.onekp.com/public_data.html
*Mesotaenium kramstei* UTEX LB 1025 transcriptome, 1KP Code NBYP	Carpenter et al.^50^	http://www.onekp.com/public_data.html
*“Mesotaenium endlicherianum“* SAG12.97 transcriptome, 1KP Code WDCW	Carpenter et al.^50^	http://www.onekp.com/public_data.html
*Mesostigma viride* CCAC 1140 transcriptome	Ju et al.^59^	https://www.ncbi.nlm.nih.gov/Traces/wgs/wgsviewer.cgi?val=GBSK00000000
*Mesostigma viride* NIES995 transcriptome	de Vries et al.^58^	https://www.ncbi.nlm.nih.gov/Traces/wgs/wgsviewer.cgi?val=GFXX00000000
*Micrasterias fimbriata* ASW 07026 transcriptome, 1KP Code MCHJ	Carpenter et al.^50^	http://www.onekp.com/public_data.html
*Micromonas pusilla* genome v3.0	Worden et al.^61^	https://phytozome.jgi.doe.gov/pz/portal.html#!info?alias=Org_MpusillaCCMP1545
*Micromonas* sp. RCC299 genome v3.0	Worden et al.^61^	https://phytozome.jgi.doe.gov/pz/portal.html#!info?alias=Org_MspRCC299
*Mougeotia scalaris* SAG164.80 transcriptome	Cooper and Delwiche^53^	https://figshare.com/articles/dataset/Green_algal_transcriptomes_for_phylogenetics_and_comparative_genomics/1604778
*Mougeotia* sp. MZCH240 transcriptome	de Vries et al.^16^; Fürst-Jansen et al.^17^	https://www.ncbi.nlm.nih.gov/Traces/wgs/wgsviewer.cgi?val=GHUK00000000.
*Mougeotiopsis calospora* transcriptome assembly	This study	GenBank: GJZN00000000.1
*Mougeotiopsis calospora* transcriptome reads	This study	Sequence Read Archive: SRR19751296
*Nephroselmis pyriformis* CCMP 717 transcriptome	Cooper and Delwiche^53^	https://figshare.com/articles/dataset/Green_algal_transcriptomes_for_phylogenetics_and_comparative_genomics/1604778
*Netrium digitus* CCAC 0148 transcriptome, 1KP Code FFGR	Carpenter et al.^50^	http://www.onekp.com/public_data.html
*Nitella mirabilis* transcriptome	Ju et al.^59^	https://www.ncbi.nlm.nih.gov/Traces/wgs/wgsviewer.cgi?val=GBST01&search=GBST01000000&display=scaffolds
*Nitella mirabilis* transcriptomes of lower and upper tissues	Cooper and Delwiche^53^	https://figshare.com/articles/dataset/Green_algal_transcriptomes_for_phylogenetics_and_comparative_genomics/1604778
*Nucleotaenium eifelense* M3006 transcriptome, 1KP Code KMNX	Carpenter et al.^50^	http://www.onekp.com/public_data.html
*Oedogonium cardiacum* UTEX LB40 transcriptome	Cooper and Delwiche^53^	https://figshare.com/articles/dataset/Green_algal_transcriptomes_for_phylogenetics_and_comparative_genomics/1604778
*Oltmansiellopsis unicellularis* SCCAP K-0250 transcriptome	Cooper and Delwiche^53^	https://figshare.com/articles/dataset/Green_algal_transcriptomes_for_phylogenetics_and_comparative_genomics/1604778
*Onychonema laeve* CCAC 0151 transcriptome, 1KP Code GGWH	Carpenter et al.^50^	http://www.onekp.com/public_data.html
*Oryza sativa* Nipponbare genome v7.0	Kawahara et al.^62^	https://phytozome.jgi.doe.gov/pz/portal.html#!info?alias=Org_Osativa
*Ostreococcus lucimarinus* genome v2.0	Palenik et al.^63^	https://phytozome.jgi.doe.gov/pz/portal.html#!info?alias=Org_Olucimarinus
*Penium exiguum* CCAC 0142 transcriptome, 1KP Code YSQT	Carpenter et al.^50^	http://www.onekp.com/public_data.html
*Penium margaritaceum* SAG22.82 transcriptome	Cooper and Delwiche^53^	https://figshare.com/articles/dataset/Green_algal_transcriptomes_for_phylogenetics_and_comparative_genomics/1604778
*Phymatodocis nordstedtiana* SVCK 327 transcriptome, 1KP Code RPQV	Carpenter et al.^50^	http://www.onekp.com/public_data.html
*Physcomitrium patens* genome v3.3	Lang et al.^64^	https://phytozome.jgi.doe.gov/pz/portal.html#!info?alias=Org_Ppatens
*Picea abies* genome	Nystedt et al.^65^	https://plantgenie.org/FTP?dir=Data%2FConGenIE%2FPicea_abies%2Fv1.0
*Planotaenium ohtanii* M2697 transcriptome, 1KP Code SNOX	Carpenter et al.^50^	http://www.onekp.com/public_data.html
*Pleurotaenium trabecula* CCAC 0163 transcriptome, 1KP Code MOYY	Carpenter et al.^50^	http://www.onekp.com/public_data.html
*Salvinia cucullata* genome	Li et al.^49^	https://www.fernbase.org
*Selaginella moellendorffii* genome	Banks et al.^66^	https://phytozome-next.jgi.doe.gov/info/Smoellendorffii_v1_0
*Sphagnum fallax* v0.5 genome	Obtained from Phytozome with permission	https://phytozome-next.jgi.doe.gov/info/Sfallax_v0_5
*Spirogloea muscicola* CCAC 0214 transcriptome, 1KP Code TPHT	Carpenter et al.^50^	http://www.onekp.com/public_data.html
*Spirogyra pratensis* MZCH10213 transcriptome	de Vries et al.^16^	https://www.ncbi.nlm.nih.gov/Traces/wgs/wgsviewer.cgi?val=GICF00000000.
*Spirogyra pratensis* UTEX 921 transcriptome	Cooper and Delwiche^53^	https://figshare.com/articles/dataset/Green_algal_transcriptomes_for_phylogenetics_and_comparative_genomics/1604778
*Spirogyra pratensis* UTEX 928 transcriptome	Ju et al.^59^	https://www.ncbi.nlm.nih.gov/Traces/wgs/wgsviewer.cgi?val=GBSM01000000
*Spirogyra* sp. M1810 transcriptome, 1KP Code HAOX	Carpenter et al.^50^	http://www.onekp.com/public_data.html
*Spirogyra* sp. Transcriptome Au1	Cooper and Delwiche^53^	https://figshare.com/articles/dataset/Green_algal_transcriptomes_for_phylogenetics_and_comparative_genomics/1604778
*Staurastrum sebaldi* M1129 transcriptome, 1KP Code ISHC	Carpenter et al.^50^	http://www.onekp.com/public_data.html
*Staurodesmus convergens* M2558 transcriptome, 1KP Code WCQU	Carpenter et al.^50^	http://www.onekp.com/public_data.html
*Staurodesmus omearii* M0751 transcriptome, 1KP Code RPRU	Carpenter et al.^50^	http://www.onekp.com/public_data.html
*Tetraselmis striata* transcriptome	Cooper and Delwiche^53^	https://figshare.com/articles/dataset/Green_algal_transcriptomes_for_phylogenetics_and_comparative_genomics/1604778
*Tetraselmis suecica* transcriptome	Cooper and Delwiche^53^	https://figshare.com/articles/dataset/Green_algal_transcriptomes_for_phylogenetics_and_comparative_genomics/1604778
*Ulva mutabilis* genome	De Clerck et al.^67^	https://bioinformatics.psb.ugent.be/orcae/overview/Ulvmu
*Volvox carteri* genome v2.1	Prochnik et al.^68^	https://phytozome.jgi.doe.gov/pz/portal.html#!info?alias=Org_Vcarteri
*Xanthidium antilopaeum* M1229 transcriptome, 1KP Code GBGT	Carpenter et al.^50^	http://www.onekp.com/public_data.html
*Zygnema circumcarinatum* SAG698-1a transcriptome	de Vries et al.^58^	https://www.ncbi.nlm.nih.gov/Traces/wgs/wgsviewer.cgi?val=GFYA00000000
*Zygnema* sp.-B M1384 transcriptome 1KP Code WGMD	Carpenter et al.^50^	http://www.onekp.com/public_data.html
*Zygnema* sp. transcriptome 1KP Code FMRU	Carpenter et al.^50^	http://www.onekp.com/public_data.html
*Zygnemopsis* sp. CCAP 699/1 transcriptome 1KP Code MFZO	Carpenter et al.^50^	http://www.onekp.com/public_data.html

**Experimental models: Organisms/strains**		

*Mougeotiopsis calospora* MZCH580	Obtained from Microalgae and Zygnematophyceae Collection Hamburg (MZCH)	maintained at Microalgae and Zygnematophyceae Collection Hamburg (MZCH)
*Mougeotia* sp. MZCH240	Obtained from Microalgae and Zygnematophyceae Collection Hamburg (MZCH)	maintained at Microalgae and Zygnematophyceae Collection Hamburg (MZCH)
*Spirogyra pratensis* MZCH10213	Obtained from Microalgae and Zygnematophyceae Collection Hamburg (MZCH)	maintained at Microalgae and Zygnematophyceae Collection Hamburg (MZCH)
*Zygnema circumcarinatum* MZCH10230	Obtained from Microalgae and Zygnematophyceae Collection Hamburg (MZCH)	maintained at Microalgae and Zygnematophyceae Collection Hamburg (MZCH)

**Software and algorithms**		

BUSCO v.5.0.0	Seppey et al.^69^	https://busco.ezlab.org
FASTQC	Babraham Institute	www.bioinformatics.babraham.ac.uk/projects/fastqc
IQ-Tree v1.5.5 and v1.6.12	Nguyen et al.^70^	http://www.iqtree.org
MAFFT v7.310	Katoh and Standley^71^	https://mafft.cbrc.jp/alignment/software/
Phytools	Revell^72^	https://cran.r-project.org/web/packages/phytools/index.html
Posterior Mean Site Frequency Profiles	Wang et al.^73^	Implemented in IQ-Tree http://www.iqtree.org
Re-routing method according to Yang 1995	Yang^74^	N/A
Trimal v1.4.rev15	Capella-Gutierrez et al.^75^	http://trimal.cgenomics.org
Transcdecoder v.5.5.0	Brian J. Haas	https://github.com/TransDecoder/TransDecoder/releases
Trimmomatic v0.36	Bolger et al.^76^	http://www.usadellab.org/cms/?page=trimmomatic

### Resource availability

#### Lead contact

Further information and requests for resources and reagents should be directed to and will be fulfilled by the lead contact, Jan de Vries (devries.jan@uni-goettingen.de).

#### Materials availability

This study did not generate new unique reagents.

#### Data and code availability

• RNA-seq data have been deposited at the NCBI under the BioProject accession PRJNA849386 and the Sequence Read Archive (SRA) under the accession SRR19751296; all data are publicly available as of the date of publication. Accession numbers are additionally listed in the key resources table.
• A transcriptome assembly has been deposited at NCBI Transcriptome Shotgun Assembly Sequence Database (TSA) under the accession GJZN00000000. The version described in this paper is the first version, GJZN01000000. The assembly is publicly available as of the date of publication. The accession number is additionally listed in the key resources table. The alignment has been uploaded to Zenodo: https://doi.org/10.5281/zenodo.6805950
• No original code was used; all computational analyses were performed with published tools and are cited in the STAR Methods section.

### Experimental model and subject details

#### Algal strains

*Mougeotiopsis calospora* (strain MZCH580), *Mougeotia* sp. (MZCH240), *Spirogyra pratensis* (strain MZCH20213) and *Zygnema circumcarinatum* (MZCH10230) were obtained from the Microalgae and Zygnematophyceae Collection Hamburg (MZCH)^77^^,^^78^ and grown in WHM medium^79^ or Waris-H medium^80^ at 20°C and under full-spectrum fluorescent lamps or white LEDs (30-50 μmol photons m^-2^ s^-1^; 16h:8h light-dark cycle), if not stated otherwise in the experimental details (see below).

### Method details

#### Rationale for the application of the name *Mougeotiopsis calospora* to strain MZCH580

In terms of its gross morphology, strain MZCH580 resembles members of the genera *Klebsormidium* (Klebsormidiophyceae), *Ulothrix* (Ulvophyceae) and *Mougeotia* (Zygnematophyceae), all of which form unbranched filaments and have plate-like or parietal plastids. However, the absence of pyrenoids in strain MZCH580 is a major distinguishing character, as algae from the three mentioned genera (and classes) typically have prominent pyrenoids surrounded by a sheath of starch grains. There are, however, two historical descriptions from the late 19^th^ century that describe pyrenoid-lacking, filamentous green algae with plate like chloroplasts: *Mougeotiopsis calospora* Palla, 1894 and *Mesogerron fluitans* Brand, 1899. *Mougeotiopsis* is a putative zygnematophyte, as scalariform conjugation and the formation of zygospores was clearly documented.^9^ Instead, *Mesogerron* was only described on the basis of vegetative material, and first suspected to be related to *Ulothrix* (Ulvophyceae, Chlorophyta). Based on the marked resemblance in their vegetative characters (filament width of 15–18 μm, cell architecture, and chloroplast morphology), *Mougeotiopsis* and *Mesogerron* were later treated as heterotypic synonyms (Krieger, 1941^81^). Strain MZCH580 matches both descriptions concerning the varying cell length (including cells that are shorter than wide), cell architecture (plastid-associated nucleus) and chloroplast morphology (plate-like to parietal with pronounced lateral indentations), but it has somewhat smaller cells (filament width of 10–15 μm). The morphological similarity, however, is compelling, and variation in filament width is known for many closely related strains or species of green algae. We were unable to locate the type material of *Mougeotiopsis calospora*, but studied original material of *Mesogerron fluitans* (collected by F. Brand in 1899 and provided by the Herbarium of the Academy of Natural Sciences of Philadelphia). The dried filaments of that species were morphologically similar to those of strain MZCH580, especially in the marked variation in cell length observed in the filaments (Figure S4). Amplification of genetic material from this sample did not work.

#### Rationale for establishing a new order, Serritaeniales ord. nov.

In our phylogeny, the branch in question comprises three distinct groups of organisms: *Mougeotiopsis calospora* (one strain known), the genus *Serritaenia* (several strains known^25^), and strain SAG 12.97, a unicellular zygnematophyte that is often referred to as “*Mesotaenium endlicherianum*”. Currently, there is only one existent ordinal name that is based on the mentioned taxon names, namely Mesotaeniales Fritsch. However, the phylogenetic position of the genus *Mesotaenium* is still uncertain, as the designation of strain SAG 12.97 is potentially based on misidentification. In the opinion of some authors (S.H. and A.B.), the morphology of SAG 12.97 does not conform with the description of the type species *M. endlicherianum* Nägeli. This problem was already recognized by other specialists for zygnematophycean algae who studied strain SAG 12.97.^82^^,^^83^ Hence, we are hesitant to reuse the name Mesotaeniales and instead introduce a new ordinal name based on the well-studied and credible genus *Serritaenia*. Descriptions of the zygnematophycean orders defined in this study are provided in Table 1.

#### Light microscopy, time-lapse photography, and confocal imaging

High-resolution imaging of *Mougeotiopsis calospora* was done with the Zeiss IM35 inverted microscope (Carl Zeiss, Oberkochen, Germany) equipped with the objective lens Planapochromat 63×/1.4, electronic flash, and the Canon EOS 6D digital single-lens reflex camera (Canon, Tokyo, Japan). Time lapse imaging was performed on a Leica DM5000B microscope (Leica Microsystems Wetzlar GmbH, Wetzlar, Germany) controlled by the Micromanager software at six frames per minute, shown as 10 FPS. Color balance and contrast of micrographs were adjusted with Photoshop CS4 (Adobe Inc., CA, USA). Confocal laser scanning microscopy was done with a Leica TCS SPE system (SP5) and the Leica LCS software (Leica Microsystems Wetzlar GmbH, Wetzlar, Germany). Chlorophyll was excited with a wavelength of 635 nm and the emission of 646–782 nm was recorded. Confocal z stacks were processed and converted to three-dimensional data with the image processing package Fiji.^84^

#### Transmission electron microscopy

Algal filaments were fixed with 2 % glutaraldehyde in 75 mM cacodylate buffer (pH 7.0) for 1 h at RT, rinsed with 75 mM cacodylate buffer, and postfixed with 1 % osmium tetroxide in 75 mM cacodylate buffer overnight at 4 °C. After rinsing in cacodylate buffer, the samples were dehydrated in a graded acetone series and embedded according to Spurr.^85^ The resulting TEM blocks were sectioned on an Ultracut E ultramicrotome (Leica-Reichert-Jung, Vienna, AU), stained with 2 % uranyl acetate and 2 % lead citrate. Sections were then examined with the LEO 906E transmission electron microscope (LEO, Oberkochen, Germany) and imaged with a MultiScan Typ 794 CCD camera and the Digital Micrograph 3.4.4 software (both Gatan Inc., Pleasanton, USA).

#### RNA isolation, sequencing and phylogenomics

For the isolation of total RNA, *Mougeotiopsis calospora* was grown on a modified freshwater F/2 medium^86^ with 1% agar at 22°C. An LED light source provided photosynthetically active radiance at 120 μmol photons^∗^m^-2∗^s^-1^ under a 12:12 h light/dark photocycle. Harvesting, RNA extraction and transcriptome sequencing was carried out as described by de Vries et al.^16^ In brief, filaments of a growing algal culture were harvested and directly transferred into Trizol (Thermo Fisher, Waltham, MA, USA). The algal sample was homogenized using a Tenbroek tissue homogenizer and all following steps were performed in accordance to the manufacturer’s instructions. To remove possible residual DNA, RNA samples were treated with DNAse I (Thermo Fisher). Adequate RNA quality was verified using a formamide agarose gel. Samples were shipped on dry ice to Genome Québec (Montreal, Canada), where additional RNA quantification and quality assessments were performed using a Bioanalyzer (Agilent Technologies Inc., Santa Clara, CA, USA). Library construction was performed using the NEB mRNA stranded Library preparation kit (New England Biolabs, Beverly, MA, USA). Sequencing of the libraries was carried out on the NovaSeq 6000 (Illumina), yielding 28188133 paired end reads of 101 base pairs in length. Quality of the reads was assessed using FastQC version 0.11.7. Reads were trimmed using Trimmomatic version 0.36^76^, applying settings for quality trimming and adapter removal (ILLUMINACLIP:Adapters.fa:2:30:10:2:TRUE HEADCROP:10 TRAILING:3 SLIDINGWINDOW:4:20 MINLEN:36). The transcriptome was assembled de novo with Trinity.^87^ Transcriptome completeness was assessed with BUSCO v.5.0.0^69^ using the viridiplantae_odb10 database in the transcriptome mode. Open reading frames (ORFs) were predicted with Transdecoder v.5.5.0.

We downloaded 83 transcriptomes and genomes of Streptophyta and Chlorophyta (see key resources table). Using a previously constructed phylogenomic dataset, we searched the selected sequencing data for orthologs of the 351 highly conserved proteins.^88^ After alignment and trimming using MAFFT v7.310^71^ and trimal v1.4.rev15,^75^ careful inspection of single-protein phylogenies estimated with IQ-TREE v1.5.5 under the LG4X model was undertaken to remove contaminants and paralogs. Once the data set was refined, orthologs that were missing in over 50% of taxa were removed; that said, we retained all orthologs that were present in *Mougeotiopsis* (overwriting the aforementioned 50% filtering). We estimated a maximum likelihood phylogeny based on the concatenated alignment of a final set of 326 translated proteins (cumulative maximum of 115,424 sites; see alignment on Zenodo, https://doi.org/10.5281/zenodo.6805950;) the final set of proteins/protein-coding genes was: AAP, ABHD13, Actin, ADK2, AGB1, AGX, AKTIP, ALG11, ALIS1, AMP2B, AOAH, AP1S2, AP3M1, AP3S1, AP4M, AP4S1, APBLC, ar21, arf3, ARL6, ARP2, ARP3, arpc1, ARPC4, ATEHD2, ATG2, atp6, ATP6V0A1, ATP6V0D1, ATPDIL14, ATSAR2, Atub, BAT1, Btub, C16orf80, C22orf28, C3H4, calr, capz, CC1, CCDC113, CCDC37, CCDC40, CCDC65, cct-A, cct-B, cct-D, cct-E, cct-G, cct-N, cct-T, cct-Z, CDK5, CLAT, COP-beta, COPE, COPG2, COPS2, COPS6, COQ4-mito, CORO1C, crfg, CRNL1, CS, CTP, D2HGDH-mito, DCAF13, DHSA1, DHSB3, DHYS, DIMT1L, DNAI2, DNAJ, DNAL1, DNM, DPP3, DRG2, ECHM, EF2, EFG-mito, EFTUD1, EIF3B, EIF3C, EIF3I, EIF4A3, EIF4E, ERLIN1, ETFA, FA2H, FAH, FAM18B, FAM96B, FAM, fh, fibri, FOLD, fpps, FTSJ1, GAS8, GCST, gdi2, GDI, glcn, GLGB2, GMPP3, gnb2l, gnbpa, GNL2, grc5, GRWD1, GSS, Gtub, H2A, H2B, h3, h4, HDDC2, HGO, HM13, hmt1, HSP70C, hsp70mt, HSP90, HYOU1, if2b, if2g, if2p, if6, IFT46, IFT57, IFT88, IMB1, IMP4, ino1, IP5PD, IPO4, IPO5, KARS, KDELR2, l10a, l12e-D, LRRC48, mat, mcm-A, mcm-B, mcm-C, mcm-D, mcm-E, metap2, METTL1, MLST8, MMAA-mito, mra1, MTHFR, MTLPD2, MYG1, NAA15, NAE1, NAPA, ndf1, NDUFV2-mito, NFS1-mito, NMD3, NMT1, NOP5A, NSA2, nsf1-C, nsf1-E, nsf1-G, nsf1-H, nsf1-I, nsf1-J, nsf1-K, nsf1-L, nsf1-M, nsf2-A, nsf2-F, ODB2, ODBA, ODBB, ODO2A, ODPA2, ODPB, oplah, orf2, osgep, PABPC4, pace2-A, pace2B, Pace2C, pace5, PCY2, PELO, PGM2, PIK3C3, PLS3, PMM2, PMPCB, PPP2R3, PPP2R5C, PPX2, PR19A, PSD11, PSD7, psma-A, psma-B, psma-C, psma-E, psma-F, psma-G, psma-H, psma-J, psmb-K, psmb-L, psmb-M, psmb-N, PSMD12, PSMD6, psmd, PURA, PYGB, rac, rad23, Rad51A, ran, RBX1, rf1, rla2a, rla2b, RPAC1, RPF1, rpl11, rpl12, Rpl13A, Rpl13e, Rpl14e, Rpl15, rpl17, Rpl18, rpl19, rpl20, rpl21, Rpl24A, rpl26, rpl27, Rpl2, rpl30, rpl31, rpl32, rpl33, rpl35, Rpl3, rpl43, rpl44, Rpl4b, Rpl5, rpl6, Rpl7a, rpl9, RPN1B, rpo-A, rpo-B, rpo-C, RPPK, rppO, rps10, rps11, rps12, rps14, rps15, rps16, rps17, rps18, rps20, rps23, rps26, rps27, rps2, rps3, rps4, rps5, rps6, rps8, RPTOR, RRAGD, RRM1, s15a, s15p, sap40, SCO1-mito, SCSB, SEC23, SF3B2, SND1, SPTLC1, sra, srp54, STXBP1, suca, SYGM1, SYNJ, tfiid, TM9SF1, TMS, topo1, trs, UBA3, ubc, UBE12, UBE2J2, Ubq, VAPA, VARS, vata, vatb, vatc, vate, VBP1, VPS18, VPS26B, WBSCR22, WD66, wd, wrs, xpb, YKT6. This tree was used as a guide to infer the final phylogeny under the LG+PMSF(C60)+F+Γ model^73^ of evolution; this is in line with the results of ModelFinder,^89^ which determined from 144 protein models LG+F+I+G4 as best-fit model according to Bayesian Information Criterion. Bootstrap analysis was conducted with 100 nonparametric bootstrap replicates using this model.

#### Ancestral character state reconstruction

Ancestral character state reconstruction was performed with Phytools (Revell^72^), which implements Yang’s^74^ re-rooting method to infer marginal ancestral state estimates for the internal nodes in the tree (Figure 3). We performed two independent analyses assuming 2-, and 4-character states in order to understand the effect of character coding on the inferred ancestral character states. The 2-state model used (1) unicellular and (2) multicellular *sensu lato* (filamentous or multicellular); the 4-state model differentiated between (2) *bona fide* filamentous algae excluding desmids, (3) chain-like filamentous desmids, and (4) multicellular *sensu stricto* (embryophytes, Coleochaetophyceae, Charophyceae, *Volvox, Ulva*). All models assumed unordered states (equal rates of change).

### Quantification and statistical analysis

For the quantification of the average diameter of macrotubules, 446 sections of macrotubules were examined with the LEO 906E transmission electron microscope (LEO, Oberkochen, Germany) and imaged with a MultiScan Typ 794 CCD camera; all 446 counts of the diameter were obtained with the Digital Micrograph 3.4.4 software (both Gatan Inc., Pleasanton, USA).

After inspection of single-protein phylogenies estimated with IQ-TREE v1.5.5 under the LG4X to remove contaminants and paralogs, the data set was refined: orthologs that were missing in over 50% of taxa were removed; that said, we retained all orthologs that were present in *Mougeotiopsis* (overwriting the 50% filtering). The final phylogeny was inferred under the LG+PMSF(C60)+F+Γ model^73^ of evolution; this is in line with the results of ModelFinder,^89^ which determined from 144 protein models LG+F+I+G4 as best-fit model according to Bayesian Information Criterion. Bootstrap analysis was conducted with 100 nonparametric bootstrap replicates using this model; approximate likelihood ratio test (SH-aLRT) was carried out with 1000 replicates and additionaly approximate Bayes (aBayes) test was carried out.

For the Approximately Unbiased test of the phylogenetic tree, we compared our phylogenomic hypothesis with that previously proposed by the One Thousand Plant Transcriptomes Initiative^6^ (main ASTRAL tree in Figure 2 based on 410 loci), which differed with ours in the relative position of a few species within Desmidiales. We performed an Approximately Unbiased test (AU test)^90^ under best-fit LG+C60+F+Γ model with 10,000 multiscale bootstrap replicates using IQ-TREE v.1.6.12.

## Supplemental information

Document S1. Figures S1–S4

Document S2. Article plus supplemental information
