# Supplementary material for: A phylogenomically informed five-order system for the closest relatives of land plants
Source: Curr Biol. 2022 Oct 24;32(20):4473–4482.e7. doi: 10.1016/j.cub.2022.08.022 (PMC9632326; doi:10.1016/j.cub.2022.08.022)
Supplement: Document S1. Figures S1–S4 [file mmc1.pdf]

Current Biology, Volume *32*

Supplemental Information

A phylogenomically informed five-order system for the closest relatives of land plants

Sebastian Hess, Shelby K. Williams, Anna Busch, Iker Irisarri, Charles F. Delwiche, Sophie de Vries, Tatyana Darienko, Andrew J. Roger, John M. Archibald, Henrik Buschmann, Klaus von Schwartzenberg, and Jan de Vries

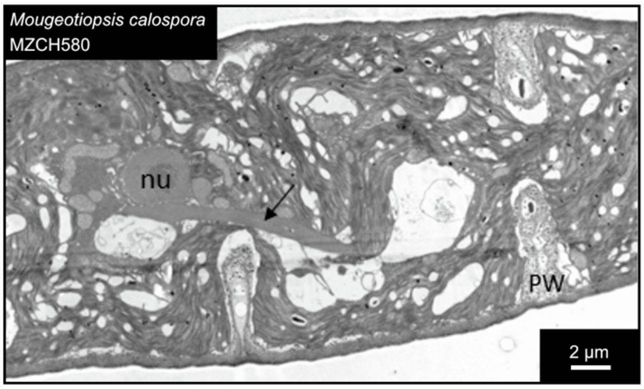

Figure S1: Macrotubule formation as detected in TEM sections of *Mougeotiopsis calospora* cells with incomplete cytokinesis, related to Figure 1. Overview showing three cells with incomplete cytokinesis and partial cross walls. Arrow indicates bundle of macrotubules.

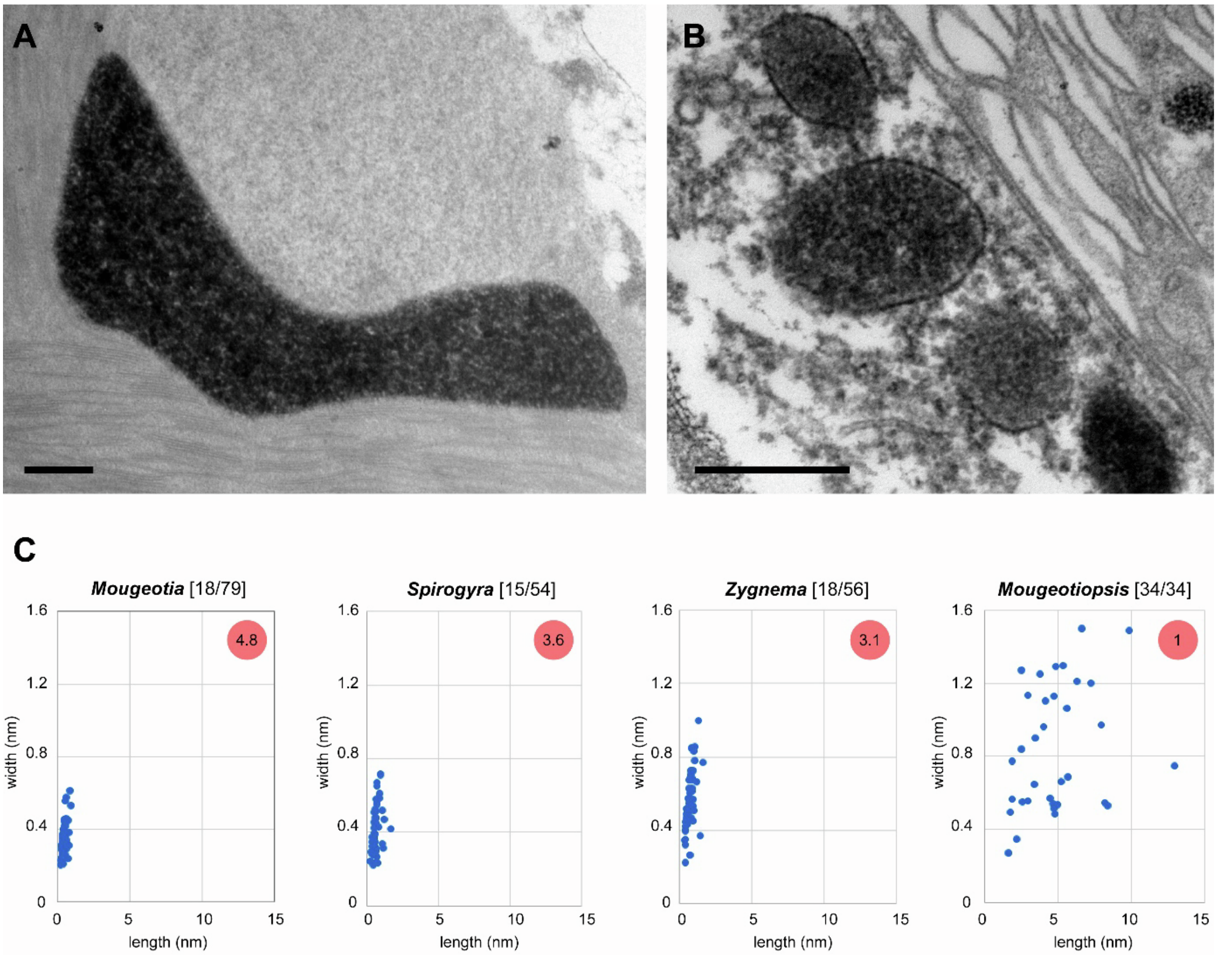

Figure S2: Peroxisomes of filamentous zygnematophytes, related to Figure 1. A: Transmission electron micrograph of a DAB-stained peroxisome of *Mougeotiopsis calospora*, strain MZCH580. **B:** Transmission electron micrograph of DAB-stained peroxisomes of *Mougeotia* sp., strain MZCH240. **C:** Sizes of peroxisome sections of four filamentous zygnematophytes as measured in transmission electron micrographs. The number of analysed cells/peroxisomes are shown in square brackets, and the average number of peroxisome cross sections per cell in the red circles. Scale bars, 0.5 µm.

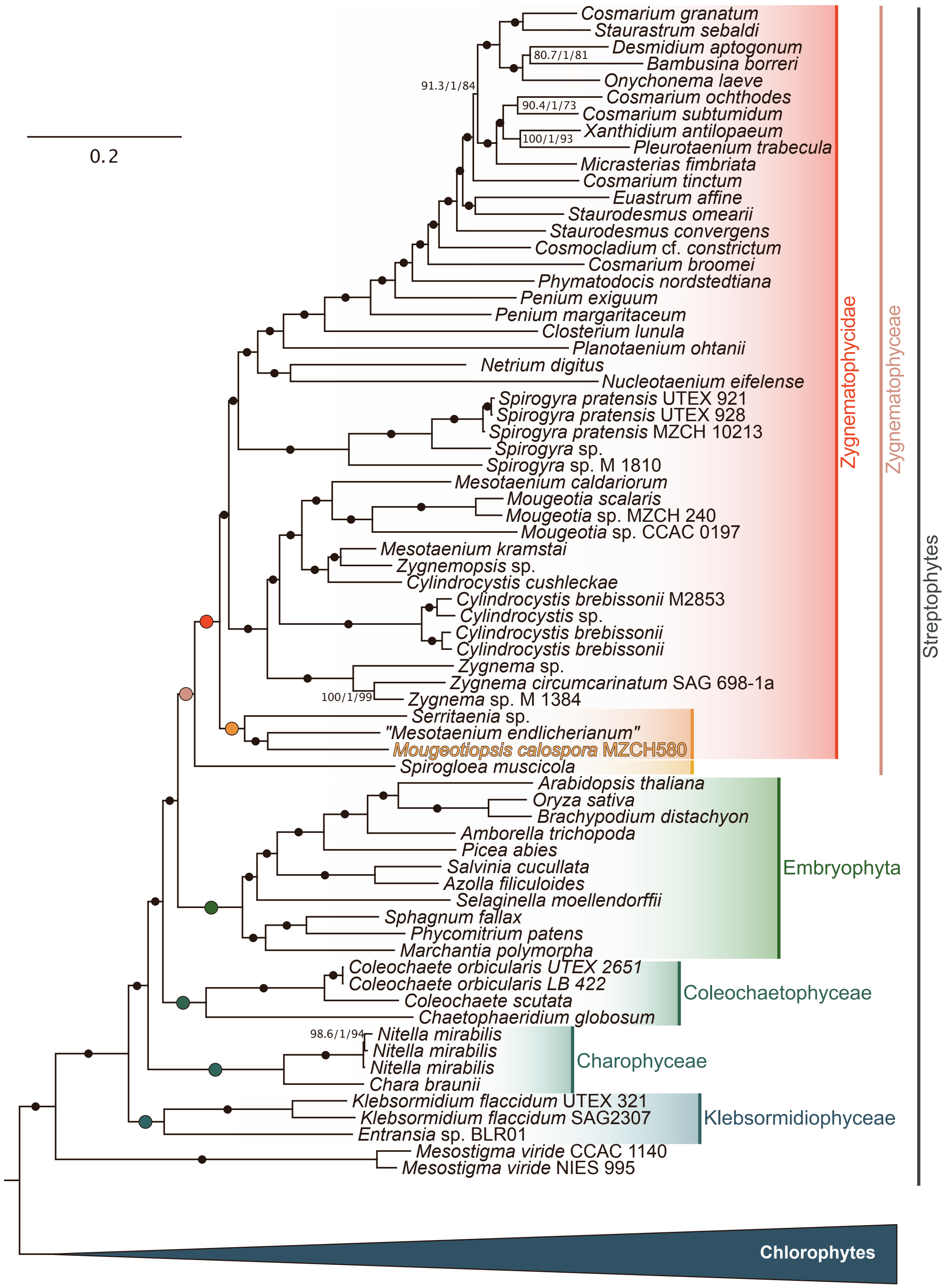

Figure S3: Multigene phylogeny of 84 Viridiplantae, related to Figure 2. Phylogenomic tree that shows the relationship of all streptophyte species analysed; the tree was rooted with the clade of chlorophytes. Scale bar, 0.2 substitutions per site. Support values from three analyses (SH-aLRT/aBayes/nonparametric bootstrapping) are shown at the corresponding branches, except for branches with maximum support (marked by dots); colored dots correspond to the (full) support recovered for the higher-order clades labeled on the right.

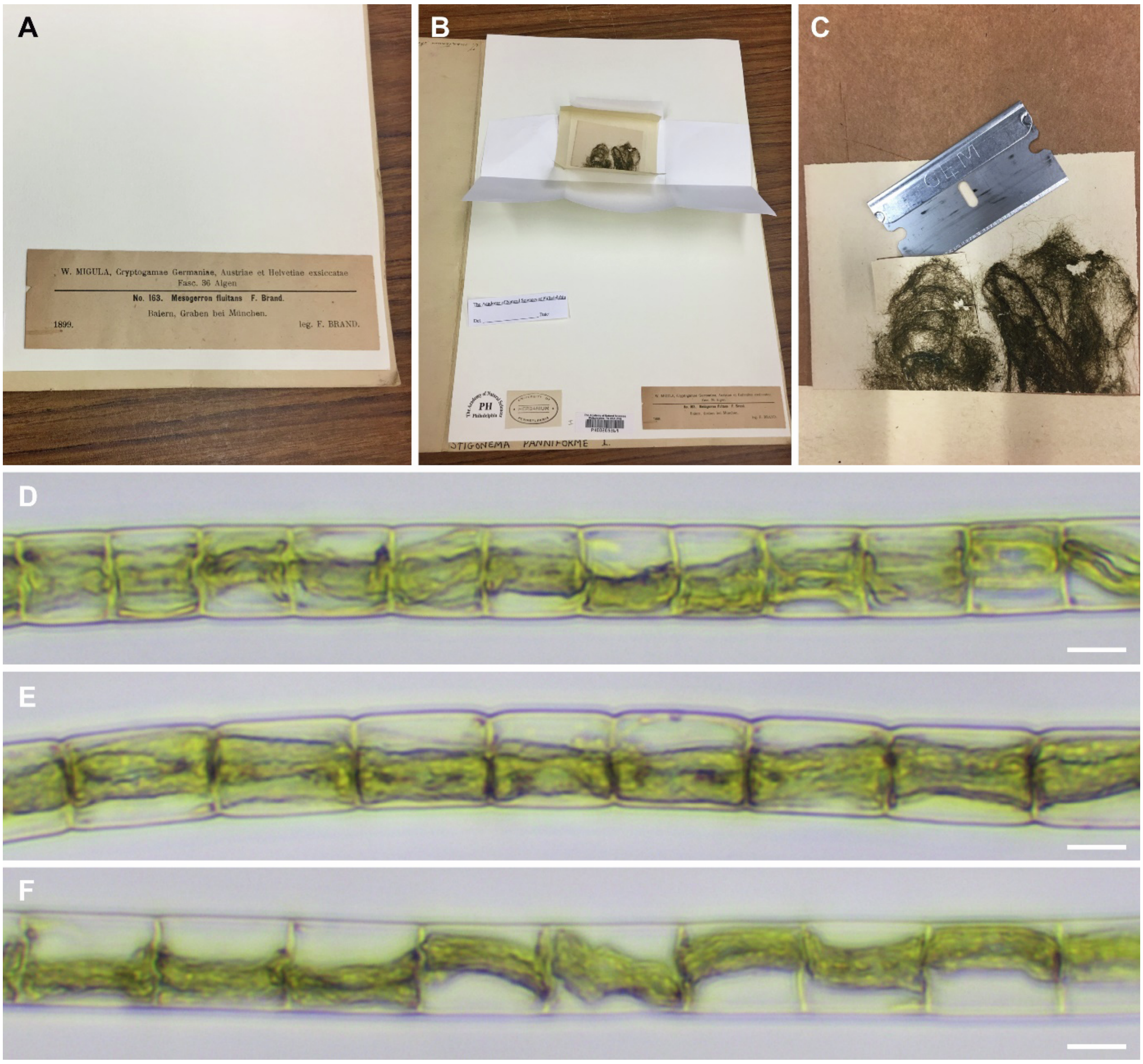

Figure S4: Destructive sampling of *Mesogerron fluitans* collected by Brand and morphological characteristics of the material, related to Figure 1. A and B: Specimen in the Herbarium of the Academy of Natural Sciences of Philadelphia (PH). **C:** Removal of dried algal material. **D–F:** Rehydrated algal filaments of the sample. Note the varying cell length and the chloroplast morphology resembling that of strain MZCH580. Images in A–C: courtesy of Richard McCourt. Scale bars, 10 µm.
